# Supplementary material for: A Global Screen for Assembly State Changes of the Mitotic Proteome by SEC-SWATH-MS
Source: Cell Syst. 2020 Feb 26;10(2):133–155.e6. doi: 10.1016/j.cels.2020.01.001 (PMC7042714; doi:10.1016/j.cels.2020.01.001)

Q5VZL5 | ZMYM4_HUMAN | ZMYM4 KIAA0425 ZNF262
Monomer MW [kDa]: 172.788 Monomer expected elution fraction: 35

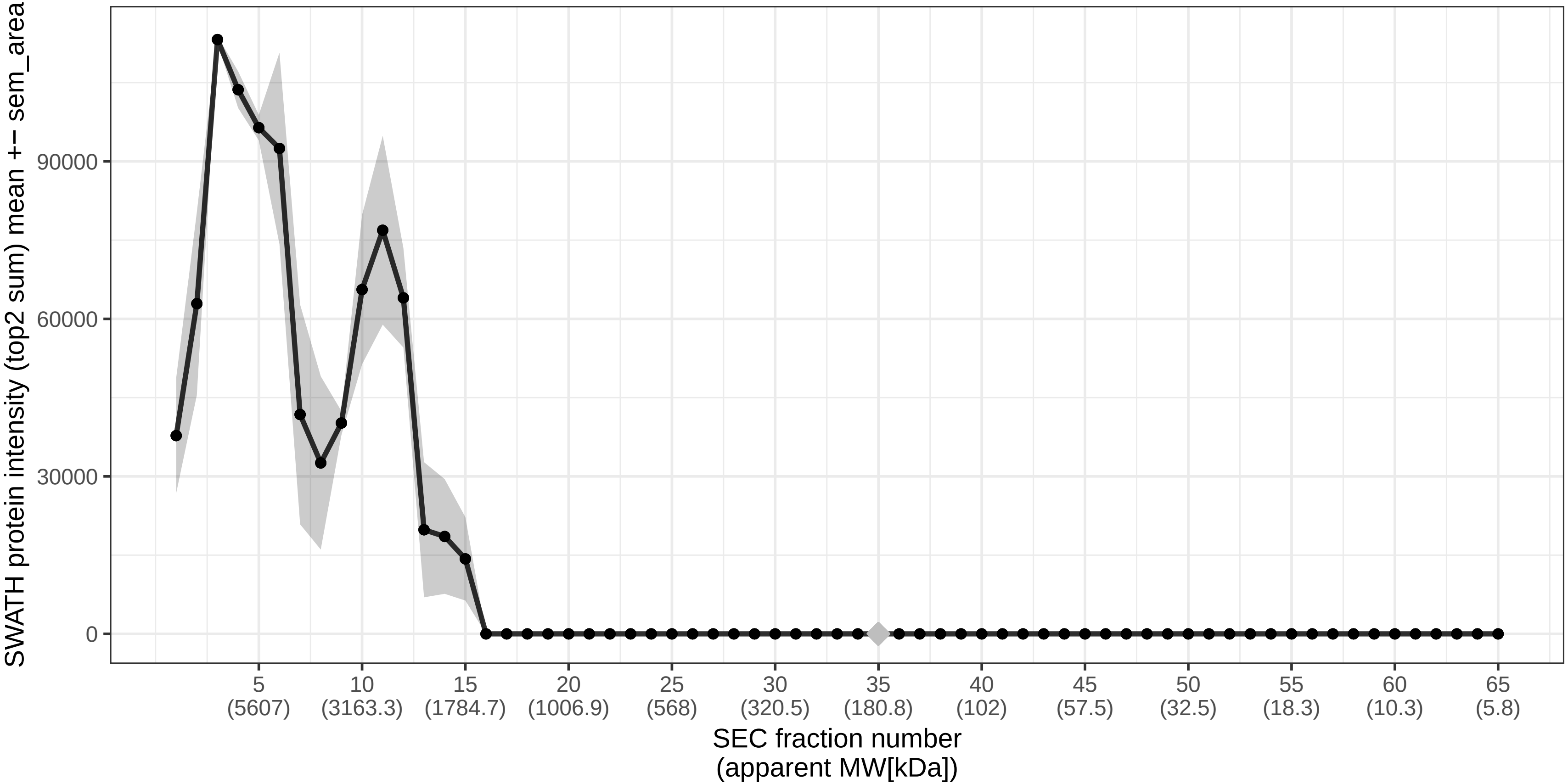

Supplement: Data S1. SEC-SWATH-MS Protein Chromatograms, Related to Figure 1 [file mmc6.zip › SECchrom_Q5VZL5_ZMYM4_HUMAN_ZMYM4_KIAA0425_ZNF262.pdf]
